# Nutritional and Supplementation Strategies to Prevent and Attenuate Exercise-Induced Muscle Damage: a Brief Review

**DOI:** 10.1186/s40798-018-0176-6

**Published:** 2019-01-07

**Authors:** Patrick S. Harty, Megan L. Cottet, James K. Malloy, Chad M. Kerksick

**Affiliations:** Exercise and Performance Nutrition Laboratory, School of Health Sciences, Lindenwood University, St. Charles, MO 63301 USA

**Keywords:** EIMD, Muscle damage, Soreness, Supplementation, DOMS, Nutrition, Recovery, Performance, Exercise

## Abstract

Exercise-induced muscle damage (EIMD) is typically caused by unaccustomed exercise and results in pain, soreness, inflammation, and reduced muscle function. These negative outcomes may cause discomfort and impair subsequent athletic performance or training quality, particularly in individuals who have limited time to recover between training sessions or competitions. In recent years, a multitude of techniques including massage, cryotherapy, and stretching have been employed to combat the signs and symptoms of EIMD, with mixed results. Likewise, many varied nutritional and supplementation interventions intended to treat EIMD-related outcomes have gained prominence in the literature. To date, several review articles have been published that explore the many recovery strategies purported to minimize indirect markers of muscle damage. However, these articles are very limited from a nutritional standpoint. Thus, the purpose of this review is to briefly and comprehensively summarize many of these strategies that have been shown to positively influence the recovery process after damaging exercise. These strategies have been organized into the following sections based on nutrient source: fruits and fruit-derived supplements, vegetables and plant-derived supplements, herbs and herbal supplements, amino acid and protein supplements, vitamin supplements, and other supplements.

## Key Points

- Exercise-induced muscle damage typically results in impaired performance, increased pain and soreness, and reduced training quality. Athletes and active individuals who require rapid recovery between bouts of damaging exercise/physical activity should implement well-supported nutritional and supplementation strategies to augment and assist with the recovery process.
- A wide assortment of nutritional and supplementation strategies have been investigated by researchers, with varying results. Initial evidence suggests that the long-term consumption of antioxidant-rich foods (tart cherry juice, pomegranate juice, beetroot juice, and watermelon juice) as well as several chronic supplementation strategies (creatine, omega-3 polyunsaturated fatty acids, and vitamin D_3_) may help to reduce symptoms of exercise-induced muscle damage and improve muscle function in a variety of populations.
- Further information is required regarding the potency of these strategies in female populations as well as the efficacy of many promising supplements such as pineapple-derived proteases, ginger, ginseng, curcumin, taurine, β-hydroxy-β-methylbutyrate, and caffeine.

## Background

Exercise-induced muscle damage (EIMD) is a transient phenomenon caused by unaccustomed, damaging exercise and is characterized by structural damage to myofibers and secondary inflammation resulting from leukocyte infiltration into the damaged tissues [1–3]. Signs and symptoms of EIMD often persist for several days after cessation of exercise and typically include muscle soreness, decreased pressure pain threshold (PPT), localized swelling, temporary decrements in maximal force-generating capacity, and elevated levels of intramuscular enzymes such as creatine kinase (CK), lactate dehydrogenase (LDH), and myoglobin (MYO) [3–5]. In addition, EIMD often results in elevations in markers of inflammation such as C-reactive protein (CRP) and various interleukins [6]. Exercise-induced muscle damage has been studied for several decades and its etiology has been extensively reviewed in a wide assortment of publications over the last 30 years (refs [1, 2, 5–11]). However, caution must be exercised when modulating inflammation and oxidative stress caused by exercise, as these outcomes are known to be a key part of the adaptive remodeling process [11]. Interested readers are encouraged to examine a recent review by Owens and colleagues [11] which outlines the rationale for the application of nutritional strategies to reduce muscle damage in light of these concerns.

Nonetheless, the alleviation of symptoms of muscle damage may be advantageous to individuals who require rapid recovery between bouts of physical activity [6, 12]. Common therapeutic interventions that are used to treat the symptoms of EIMD include stretching, massage, electrotherapy, cryotherapy, non-steroidal anti-inflammatory drugs (NSAIDS), as well as nutritional and supplementation strategies [2, 6, 13]. Earlier reviews [12, 14–16] have summarized some (but not all) of the widely researched dietary and supplementation strategies that have been examined for their ability to alleviate symptoms of EIMD. In short, Bloomer [14] provided an early review covering the efficacy of antioxidants, β-hydroxy-β-methylbutyrate, as well as several other assorted supplements to reduce the severity of EIMD. Sousa et al. [12] later reviewed the physiological mechanisms of EIMD and oxidative stress as well as preliminary evidence surrounding the efficacy of protein, vitamin, polyphenol, and n-3 polyunsaturated fatty acid (n-3 PUFAs) consumption to reduce the signs and symptoms of EIMD. Similarly, Kim and colleagues [15] briefly covered the efficacy of n-3 PUFAs, caffeine, taurine, and dietary polyphenols. Finally, Köhne et al. [16] conducted a systematic review and identified five studies that examined the effect of a nutritional intervention on EIMD-related outcomes in female participants. However, none of these studies have comprehensively collated all well-supported or emerging nutritional strategies that are purported to reduce the signs and symptoms of EIMD. Furthermore, a large body of peer-reviewed literature investigating the effects of such interventions on EIMD has accumulated over the ensuing years. Because no single publication to date has comprehensively reviewed the many relevant nutritional and supplementation strategies that may reduce the magnitude of EIMD, the intent of this review is to briefly outline the efficacy of each strategy that has been suggested to alleviate exercise-induced muscle damage. It is our hope that this review will serve as a key reference in the development of future studies as scientists continue to explore nutritional means to optimize recovery and minimize negative perturbations secondary to EIMD.

Due to length restrictions, studies which employed acute damaging exercise interventions were the focus of this review, and any nutritional strategy that has not been investigated by at least two well-controlled investigations was excluded. For the purposes of this review, damaging exercise is defined as any exercise stimulus which significantly modulated a primary outcome measure associated with EIMD, such as soreness, muscle function, serum markers of muscle damage, or cytokines. Due to systemic shortcomings in a great deal of the pertinent oxidative stress literature in this area, all outcomes related to oxidative stress were excluded from the scope of this review [18]. To ensure brevity where appropriate and to reduce the number of references, several key review articles covering extensively researched nutritional adjuncts (i.e., vitamin C and E, protein, BCAAs, etc.) were cited directly. Because these recent summaries are available, very little additional discussion has been offered in these instances. Thus, this review is organized into six sections based on nutrient source: fruits and fruit-derived supplements, vegetables and plant-derived supplements, herbs and herbal supplements, amino acid and protein supplements, vitamin supplements, and other supplements. Due to length restrictions, dosing protocols and exercise intensity are typically reported in parentheses, with the latter using the following format: (sets x reps, intensity). In addition, summary tables have been included in each section that outline the mechanism of action, key nutrient, and suggested benefits of each nutritional strategy.

## Main Text

### Fruits and Fruit-Derived Supplements

#### Black Currant

Preliminary results suggest that the consumption of black currant extract (BE) may mitigate indirect markers of muscle damage. Blackcurrants are rich in anthocyanins, which are naturally occurring pigments that have potent antioxidant and anti-inflammatory properties [19, 20]. A 2009 investigation [20] demonstrated that BE supplementation (~ 48 g total, 2 h before and 2 h after exercise) significantly blunted elevations in CK induced by 30-min rowing exercise at 80% maximal oxygen uptake (VO_2max_) relative to isocaloric placebo in healthy adults who performed daily moderate physical activity. These results were mirrored by a later study [19] which found that moderately active, non-resistance-trained adults who consumed blackcurrant juice (473 mL twice daily for 8 days) prior to eccentric squat exercise (10 × 10, 115% one-repetition maximum [1RM]) performed after 4 days of supplementation displayed reduced circulating CK levels at both 48 h and 96 h after exercise compared to placebo (Table 1).

**Table 1:** Overview of fruits and fruit-derived supplements

Nutrient source (key nutrient)	Mechanism of action	Suggested benefits	Refs
Blackcurrant (*Anthocyanins*)	Antioxidant properties	↓DMG	[19, 20]
Cherries (*Anthocyanins*)	Antioxidant properties	↓DMG, ↓DOMS, ↓INF, ↑MF	[22–33]
Pineapple (*Bromelain*)	Anti-inflammatory properties	↓DOMS, ↓INF, ↑MF	[34–36, 38]
Pomegranate (*Ellagitannins*)	Antioxidant properties	↓DMG, ↓DOMS, ↑MF	[39–42]
Watermelon (*Citrulline*)	Antioxidant properties	↓DMG, ↓DOMS, ↑MF	[43–49]

*↓DMG* nutrient reduced indirect markers of muscle damage compared to placebo, *↓DOMS* nutrient reduced soreness and delayed-onset muscle soreness compared to placebo, *INF* nutrient reduced markers of inflammation compared to placebo, *↑MF* nutrient improved muscle function relative to placebo

#### Tart Cherry Juice

The chronic consumption of tart cherry juice appears to effectively modulate the symptoms of EIMD, as tart cherries are rich in anthocyanins and thus possess anti-inflammatory and antioxidant effects when consumed in appreciable doses [21]. Bowtell et al. [22] demonstrated that consumption of tart cherry concentrate (60 mL/day for 10 days) in trained males significantly increased the rate of isometric force recovery following a bout of knee extensions (10 × 10, 80%1RM) performed after 7 days of supplementation. A similar study [23] reported that EIMD-related decrements in force production and subjective pain during the 96 h after eccentric elbow exercise (2 × 20, maximal voluntary contraction [MVC]) after 3 days of supplementation were significantly reduced in college-aged males who consumed 710 mL/day tart cherry juice for 8 days. Levers et al. [24] likewise reported that supplementation (480 g/day for 10 days) with tart cherry skin powder reduced perceived soreness, serum creatinine, and serum total protein relative to placebo during the 48 h after exercise in resistance-trained males who performed barbell back squats (1 × 10, 70%1RM) after 7 days of supplementation. Chronic consumption of tart cherry juice (approx. 60 mL juice concentrate/day or 500 mL juice/day for 7–8 days) appears to also ameliorate decrements in muscle function [25–27], reduce biomarkers of EIMD [25, 28–30], and decrease perceptions of pain both immediately after [31] and during 48 h of recovery [32] after intermittent or extended endurance exercise in a variety of populations. Finally, it is important to note that the single study included in the present review which did not show an effect of tart cherry consumption on EIMD used an exercise stimulus that did not induce sufficient damage, thus rendering any potential treatment effect moot [33]. It is evident that tart cherry juice shows promise as an emerging strategy to reduce the magnitude of EIMD (Table 1).

#### Pineapple (Bromelain)

Pineapple-derived protease (bromelain) supplementation, particularly when combined with other proteolytic enzymes, has been shown to result in improved muscle function [34–36], reduced soreness [34], and higher pressure pain threshold [34] after damaging exercise compared to placebo. Protease supplementation has been suggested to inhibit the production of pro-inflammatory agents while exerting anti-inflammatory effects [34, 37]. However, the effects of these nutritional agents on biomarkers of muscle damage are less clear. Miller et al. [34] reported that healthy males who consumed a combined protease supplement (4.2 g/day for 4 days) prior to 30-min downhill running at 80% maximal heart rate (MHR) experienced faster recovery of force production, less soreness, and increased pressure pain threshold during the 72 h after exercise compared to age, height, and weight-matched counterparts who consumed placebo. These results were supported by a later study [35] which found that protease supplement consumption (5.8 g/day for 24 days) in recreationally active males who performed structured exercise at least three times per week resulted in reduced serum cytokine levels and blunted decrements in MVC caused by a bout of downhill running (45 min, 60%VO_2_max) performed after 21 days of supplementation. Udani et al. [38] reported that untrained subjects who consumed a protease-containing supplement for 30 days prior to high-volume smith machine squat exercise (participants completed twice as many repetitions as they were able to complete during a 5-min period during the initial familiarization session) reported less pain after 48 h of recovery and less tenderness 24 h after exercise relative to placebo. However, between-group differences in CK, CRP, or cytokines were not detected. Clearly, these supplements show promise, though further information is required (Table 1).

#### Pomegranate Juice

Recent research suggests that the chronic consumption of pomegranate juice may improve functional measures and reduce biomarkers of EIMD. Pomegranate juice is a rich source of ellagitannins, a type of polyphenol with antioxidant and anti-inflammatory properties [39, 40]. Trombold and colleagues [39] found that recreationally active males who consumed pomegranate juice (800 mL/day for 9 days) retained significantly greater muscle function following damaging eccentric exercise (2 × 20, MVC) on day 5 of supplementation compared to those who consumed an isocaloric placebo. These results were replicated by the same research group [40] using resistance-trained males who consumed pomegranate juice (500 mL/day) for 15 days. On the eighth day, the subjects performed 3 × 20 maximal eccentric elbow extensions and 6 × 10 maximal eccentric knee extensions. Post-exercise measures of upper body but not lower body strength were preserved and soreness decreased relative to placebo during the entire 7 days of recovery in the treatment group. The researchers hypothesized that minor differences in the exercise stimulus applied to the elbow flexors and knee extensors or the relatively greater action of the knee extensors during activities of daily living may have contributed to this pattern. A third investigation conducted by the group [41] found that once-daily consumption of 30 mL pomegranate juice concentrate had similar efficacy relative to twice-daily consumption (60 mL/day) in recreationally active men. After 3 days of supplementation, the subjects performed a 20-min bout of downhill running and 40 eccentric elbow contractions at 100%1RM. During the 4-day recovery phase, the researchers noted that both treatment groups displayed higher knee extension and elbow flexion MVC relative to placebo. A recent study [42] likewise demonstrated that elite weightlifters who consumed pomegranate juice (750 mL/day, 500 mL 1 h before exercise) in the 48 h prior to high-intensity weightlifting training reported lower subjective soreness at the 48 h time point after exercise and displayed lower post-exercise levels of CK and LDH compared to placebo (Table 1).

#### Watermelon Juice/Citrulline

The acute consumption of watermelon juice, a rich source of the amino acid l-citrulline, has been shown to improve EIMD-related outcomes, though results are mixed. l-citrulline possesses antioxidant properties and also acts as a precursor to l-arginine in the nitric oxide cycle [43]. Tarazona-Diaz et al. [43] reported that recreationally active males who consumed placebo 1 h prior to intense cycle interval sprints (8 × 30 s, 1-min rest) reported significantly more soreness after 24 h of recovery compared to males who consumed 500 mL watermelon juice (1.17 g citrulline) or 500 mL of citrulline-enriched watermelon juice (6 g citrulline). Martinez-Sanchez and colleagues [44] found that a similar supplementation protocol of enriched watermelon juice (3.45 g citrulline) 1 h prior to a half-marathon race attenuated muscle soreness during 24–72 h of recovery and preserved muscle function relative to placebo. Moreover, the same research group [45] noted that resistance-trained subjects who consumed 200 mL of either citrulline-enriched watermelon juice (3.3 g citrulline) or ellagitannin and citrulline-enriched watermelon juice (containing 3.3 g citrulline, 22 mg ellagitannins) prior to intense half-squat exercise (8 × 8, 8RM) reported reduced soreness during 48 h of recovery, retained greater muscle function, and exhibited lower levels of MYO relative to placebo. Pérez-Guisado et al. [46] likewise found that the consumption of a citrulline-containing supplement (8 g citrulline malate) reduced subjective soreness during the 48 h after bench press exercise (eight sets to fatigue, 80%). Conversely, Shanely et al. [47] found no effect of watermelon puree consumption (980 mL/day for 14 days) on markers of inflammation in trained cyclists after a 75-km time trial. da Silva et al. [48] also reported that acute citrulline malate supplementation (6 g) did not affect subjective soreness or serum CK compared to placebo during the 72 h following damaging (a main effect for time was observed for soreness and CK) leg press and hack squat exercise (3 × 8–12, 90% 10RM, 2 s eccentric contraction). Finally, a 2018 study [49] found that acute citrulline malate supplementation (~ 4.2 g citrulline) increased perceived soreness compared to placebo during the 72-h period after isokinetic knee extensions (10 × 10, 70%MVC) in resistance-trained males and females (Table 1).

### Vegetables and Plant-Derived Foods and Supplements

#### Beetroot Juice

Recent investigations have indicated that the consumption of beetroot juice after certain types of exercise may act to reduce the magnitude of EIMD. Beetroots are rich in dietary nitrates and betalains, a type of pigment with antioxidant and anti-inflammatory properties [50]. Clifford and colleagues [50] first demonstrated that the consumption of high (1.75 L total, ~ 2.8 g phenolic compounds) and low doses (0.875 L total, ~ 1.4 g phenolic compounds) of beetroot juice in the 48 h following eccentric plyometric exercise (5 × 20 drop jumps, 0.6 m height) resulted in significantly faster recovery of PPT relative to placebo during a 72-h recovery period in recreationally active males, though muscle function was only improved relative to placebo in the high-dose group. These results were mirrored in a later study by the same research group [51], which found that consumption of beetroot juice (1.75 L total, ~ 2.8 g phenolic compounds) in the 48 h after exercise attenuated changes in PPT during the 72-h recovery period after eccentric plyometric exercise compared to a nitrate-matched drink or isocaloric placebo in recreationally active males. Beetroot juice (2 L total) consumed during the 48 h after repeated sprint exercise has also been shown to result in faster recovery of countermovement jump performance and reactive strength index scores compared to placebo [52]. Likewise, Montenegro et al. [53] demonstrated that beetroot concentrate supplementation (100 mg/day for 7 days) attenuated the creatine kinase response to a 10-km running time trial in male and female competitive triathletes. However, Clifford and colleagues [54] found no effect of beetroot juice consumption (1.75 L total, ~ 2.8 g phenolic compounds) on EIMD-associated outcomes in trained runners who followed a similar supplementation protocol after a marathon, likely because the protocol did not induce significant damage in the well-trained participants (Table 2).

**Table 2:** Overview of vegetables and plant-derived supplements

Nutrient source (key nutrient)	Mechanism of action	Suggested benefits	Refs
Beetroot (*Betalains*)	Antioxidant properties	↓DMG, ↓DOMS, ↑MF	[50–54]
Green algae extract (*Astaxanthin*)	Antioxidant properties	↓DMG	[55, 56]

*↓DMG* nutrient reduced indirect markers of muscle damage compared to placebo, *↓DOMS* nutrient reduced soreness and delayed-onset muscle soreness compared to placebo, *INF* nutrient reduced markers of inflammation compared to placebo, *↑MF* nutrient improved muscle function relative to placebo

#### Green Algae Extract/Astaxanthin

Astaxanthin is a carotenoid that has been suggested to possess potent antioxidant properties [55]. However, the efficacy of chronic astaxanthin supplementation has yet to be adequately determined. Bloomer and associates [55] found no effect of green algae-sourced astaxanthin supplementation (4 mg/day for 25 days) on functional or biochemical markers of EIMD in resistance-trained men who performed a bout of eccentric knee extensions (10 × 10, 85% eccentric 1RM) after 21 days of supplementation. It is important to note that the exercise protocol employed by the researchers was a sufficiently damaging stimulus, as a main effect for time was observed for muscle soreness, CK, and muscle function. Conversely, Djordjevic et al. [56] found that post-exercise CK levels were lower after a 2-h bout of intense sport-specific exercise in elite youth soccer players who had previously consumed astaxanthin (4 mg/day) for 90 days. Clearly, more populations must be studied before these results can be generalized further (Table 2).

### Herbs and Herbal Supplements

#### Anatabine

It appears that anatabine, a tobacco-sourced alkaloid, has little effect on functional or biochemical outcomes associated with EIMD, in spite of demonstrated anti-inflammatory effects in vivo [57]. In a series of investigations, Jenkins and colleagues [58, 59] found that the chronic consumption of anatabine (6–12 mg/day for 10 days) had no effect on pain tolerance, muscular strength, or localized swelling and actually increased serum LDH relative to placebo following damaging eccentric forearm exercise (6 × 10, MVC) in non-resistance-trained males. Regrettably, the authors did not provide an explanation for the unexpected LDH flux after anatabine supplementation. While initial results do not appear promising, more work must be done in this area to determine whether the substance has merit (Table 3).

**Table 3:** Overview of herbs and herbal supplements

Nutrient source (key nutrient)	Mechanism of action	Suggested benefits	Refs
Anatabine	Anti-inflammatory properties	NA	[58, 59]
Green tea (*Polyphenols*)	Antioxidant properties	↓DMG, ↓DOMS	[60–62, 64–67]
Curcumin (*diferuloylmethane*)	Anti-inflammatory properties	↓DMG, ↓DOMS, ↓INF, ↑MF	[68–71]
Ginger (*Gingerols*, *shogaols*)	Anti-inflammatory properties	↓DOMS, ↑MF	[73–76]
Ginseng (*Ginsenosides*)	Anti-inflammatory properties	↓DMG, ↓DOMS, ↓INF, ↑MF	[78–81]
*Phyllanthus amarus* (*Polyphenols*)	Antioxidant properties	↓DOMS	[82, 83]
*Rhodiola rosea* (*Rosavin*, *Rosin*, *Rosarin*, *Salidroside*)	Antioxidant properties	↓DMG, ↓INF	[85–87]

*↓DMG* nutrient reduced indirect markers of muscle damage compared to placebo, *↓DOMS* nutrient reduced soreness and delayed-onset muscle soreness compared to placebo, *INF* nutrient reduced markers of inflammation compared to placebo, *↑MF* nutrient improved muscle function relative to placebo, *NA* nutrient had no effect on EIMD-related outcome measures

#### Green Tea

Green tea is rich in polyphenols that exert potent antioxidant properties [60, 61]. Prophylactic consumption of polyphenols from green tea has been shown to alleviate signs and symptoms of EIMD, though results are mixed. Panza et al. [62] first demonstrated that the consumption of green tea (200 mL/day for 7 days) attenuated the CK response to four sets of bench press exercise (4–10 repetitions, 75–90%1RM) relative to control in resistance-trained males. However, treatment order was not randomized and thus these results may have been unduly influenced by the repeated bout effect [63]. Jowko and colleagues [64] reported similar findings (reduced CK response after one set of bench press and back squat repetitions to fatigue at 60%1RM) in untrained males who had supplemented with green tea extract (640 mg/day for 4 weeks) during a weight training program. Kerksick et al. [61] likewise found that non-resistance-trained males who consumed epigallocatechin gallate, a polyphenol found in green tea (1800 mg/day), for 14 days prior to damaging eccentric knee extensions (10 × 10, 60°/s) reported significantly lower levels of muscle soreness 24 h after exercise compared to placebo, though between-groups differences in LDH and CK were not detected. Herrlinger and colleagues [65] also reported that consumption of green tea polyphenols (2000 mg/day for 13 weeks) prior to downhill running (40 min, 65%VO_2_max) preserved muscle function, reduced circulating CK levels, and decreased soreness 48 h after exercise relative to placebo in recreationally active males. Conversely, several studies have shown that acute [66] and chronic [67] supplementation with tea-derived polyphenols did not attenuate the post-exercise flux of indirect markers of muscle damage after damaging resistance exercise. A later investigation [60] likewise found no effect of green tea extract supplementation (980 mg/day for 28 days) on markers of muscle damage in male sprinters who performed four repeated Wingate cycle sprints. It is important to note that the exercise stimulus resulted in increased plasma CK concentrations after 24 h of recovery in both conditions, suggesting that sufficient muscle damage was induced in the well-trained participants. In conclusion, green tea-derived polyphenols show merit as a means to reduce EIMD, though further work is required to determine optimal dosing strategies (Table 3).

#### Curcumin

Preliminary results suggest that the consumption of curcumin, a compound derived from the spice turmeric, may alleviate inflammation and soreness caused by eccentric exercise. Curcumin has been demonstrated to modulate inflammation and cytokine flux by influencing COX-2 signaling [68]. Drobnic et al. [69] first performed a pilot study which found that moderately active males who consumed curcumin (200 mg/day for 4 days) before performing downhill running after 2 days of supplementation reported less quadriceps pain 48 h after exercise and displayed significantly less muscle injury as measured by magnetic resonance imaging (MRI). Nicol and colleagues [70] likewise found that supplementation with curcumin (5 g/day) for 5 days moderately reduced post-exercise pain during 48 h of recovery and blunted the CK flux caused by 7 × 10 eccentric single-leg presses (five sets at 120%1RM, two sets at 100%1RM) in recreationally active men, though post-exercise interleukin 6 (IL-6) was elevated relative to placebo. A similar study [68] reported that CK, TNF-α, and IL-8 responses were blunted in untrained males and females who consumed curcumin (400 mg/day) for 2 days prior to and 4 days after damaging exercise (6 × 10 eccentric leg press repetitions, 110%1RM). Similarly, Tanabe and associates [71] reported a preservation of muscle function and reduced peak CK activity in untrained **s**ubjects who consumed 150 mg curcumin 1 h prior to and 12 h after eccentric elbow extensions (1 × 50, 120°/s). Clearly, curcumin shows promise as a strategy to blunt the negative effects of damaging exercise, though further information is required in a wider variety of populations (Table 3).

#### Ginger

Emerging evidence suggests that the chronic consumption of ginger may act to mitigate post-exercise soreness, though its effect on other EIMD-related outcomes is less clear. Ginger exerts analgesic effects by modulating COX signaling and inhibiting prostaglandin synthesis [72]. Black and colleagues [73] first investigated ginger in untrained participants who consumed 2 g raw or heat-treated ginger per day for 11 days. Eccentric elbow flexion exercise (3 × 6, 120%1RM) was performed on the eighth day of supplementation. The treatment groups reported less pain relative to placebo during the 24 h after exercise. However, the same research group [74] noted that an acute dose (2 g) of ginger consumed by untrained participants 24 h after eccentric elbow exercise resulted in a minor but nonsignificant reduction in arm pain during the following day relative to placebo. Interestingly, Matsumura and colleagues [75] found that male and female subjects who consumed 4 g ginger per day for 5 days prior to damaging eccentric elbow flexion exercise experienced faster recovery of elbow flexor 1RM, though CK increased during the recovery period in the treatment but not placebo group. Conversely, Wilson et al. [76] found that soreness 24 h after exercise was reduced, though muscle function unaffected in male and female trained runners who consumed 2.2 g ginger/day for 3 days before and 2 days after a 20–22 mi training run. Additional research must be conducted before results can be generalized further (Table 3).

#### Ginseng

Several studies suggest that the chronic consumption of ginseng, an herb commonly used in Eastern medicine, reduces the activity of various indirect markers of muscle damage. Ginsenosides, a class of saponins present in ginseng, have been suggested to possess immunomodulatory effects [77]. Hsu et al. [78] first demonstrated that post-exercise CK levels were blunted in male volunteers who ingested 1.6 g/day of American ginseng for 4 weeks prior to a bout of treadmill running to exhaustion (80% maximal oxygen consumption [VO_2_max]). A similar study [79] investigated the effects of supplementation with red ginseng extract (60 g/day for 7 days before and 3 days after exercise) on markers of EIMD after uphill treadmill running (2 × 45 minute bouts, 15° incline, 10 km/h). The researchers found that post-exercise CK and cytokine levels were significantly decreased relative to placebo. Pumpa et al. [80] likewise demonstrated that the consumption of 4 g Chinese ginseng 1 h before and immediately after intermittent bouts of downhill treadmill running at 80% MHR preserved muscle function, reduced soreness relative to placebo during the 96-h recovery period, and blunted the IL-6 and tumor necrosis factor-alpha (TNF-α) response compared to placebo in well-trained males. In accordance with these findings, Caldwell and colleagues [81] found that the consumption of both a high-dose (960 mg/day) and low-dose (160 mg/day) ginseng-containing supplement for 14 days prior to leg press exercise (5 × 12, 70% 1RM) reduced the change in muscle soreness during the 24-h period immediately after exercise in recreationally active males and females. While the work investigating the efficacy of ginseng as a nutritional adjunct to reduce muscle damage is preliminary in nature, it is clear that this strategy warrants further investigation (Table 3).

#### *Phyllanthus amarus*

Preliminary findings suggest that consumption of the antioxidant-rich tropical herb *Phyllanthus amarus* (PA) may attenuate muscular pain resulting from unaccustomed exercise [82]. Roengrit and colleagues [82] first investigated PA and found that post-exercise PPT was higher during the entire 48-h recovery period in untrained males who consumed supplemental PA (200 mg before and 400 mg during the day of cycling exercise, 600 mg/day during the 48 h after exercise) before and after cycling (20 min, 85% peak oxygen consumption [VO_2_peak]). However, PA supplementation had no effect on serum CK and CRP. Conversely, a later investigation by the same group [83] found that a similar dosing protocol had no effect on markers of damage, inflammation, or soreness in sedentary males who completed a 20-min bout of cycling exercise (65%VO_2_peak). While both studies reported significant post-exercise perturbations in an EIMD-related outcome, the results of these studies must be interpreted in light of the minimal nature of the exercise stimulus and untrained nature of the participants. Thus, while PA supplementation may show promise, more research is warranted (Table 3).

#### *Rhodiola rosea*

Mixed results have been reported regarding the efficacy of the adaptogenic herb *Rhodiola rosea* (RR) in reducing soreness and markers of muscle damage. This herb is rich in flavonoids and other biologically active compounds which exert powerful antioxidant properties [84]. Abidov et al. [85] first noted that consumption of RR extract (60 mg/day for 36 days) prior to a graded cycle exercise test to volitional fatigue blunted the post-exercise CK and CRP response compared to placebo and control in untrained males. Parisi et al. [86] likewise found that aerobically trained males who consumed RR extract (85 mg/day) for 4 weeks prior to a cycle test to exhaustion (75%VO_2_max) had lower post-exercise CK activity relative to placebo. Conversely, Shanely and colleagues [87] failed to demonstrate an effect of supplementation with RR extract (600 mg/day for 37 days) on markers of muscle damage induced by a marathon performed on the 30th day of supplementation in trained male and female runners. Due to the divergent results reported thus far and preliminary nature of the body of literature surrounding this strategy, more results are required before its efficacy can be determined (Table 3).

### Amino Acid and Protein Supplements

#### Branched-Chain Amino Acids

Fouré and Bendahan [88] have exhaustively examined the effects of branched chain amino acid (BCAA) supplementation on EIMD in a 2017 systematic review. In brief, the authors concluded that prophylactic BCAA supplementation of at least 200 mg/kg/day for at least 10 days may be effective to combat moderate muscle damage, though the outcomes and methodological quality of the relevant studies varied considerably. Interested readers are encouraged to consult this paper for additional discussion involving BCAA supplementation (Table 4).

**Table 4:** Overview of amino acid and protein supplements

Nutrient source (key nutrient)	Mechanism of action	Suggested benefits	Refs
BCAAs (*Leucine*, *Valine*, *Isoleucine*)	Increased MPS	↓DMG, ↓DOMS, ↑MF	[88]
Creatine	Anti-inflammatory properties	↓DMG, ↓DOMS, ↓INF, ↑MF	[89, 91–100]
HMB	Increased MPS and cellular membrane integrity	↓DMG, ↓DOMS, ↑MF	[101–106]
L-glutamine	Anti-inflammatory properties	↓DMG, ↓DOMS, ↑MF	[107–110]
Protein	Increased MPS	↓DOMS, ↑MF	[111]
Taurine	Antioxidant properties	↓DMG, ↓DOMS, ↑MF	[113–116]

*BCAAs* branched-chain amino acids, *↓DMG* nutrient reduced indirect markers of muscle damage compared to placebo, *↓DOMS* nutrient reduced soreness and delayed-onset muscle soreness compared to placebo, *↓INF* nutrient reduced markers of inflammation compared to placebo, *HMB* β-hydroxy-β-methylbutyrate, *↑MF* nutrient improved muscle function relative to placebo

#### Creatine

The results of several investigations suggest that prophylactic consumption of creatine may ameliorate the effects of EIMD, though discrepant results have been reported in a variety of populations. In addition to its role as an energy substrate, creatine has been shown to possess antioxidant and anti-inflammatory properties [89, 90]. Rawson et al. [91] first noted that creatine consumption (20 g/day for 5 days) prior to eccentric elbow flexion exercise (2 × 25, MVC) had no effect on muscle function or markers of muscle damage compared to placebo in non-resistance-trained males; results which were mirrored by a later investigation [92] which found no effect of creatine supplementation (0.3 g/kg for first 5 days, 0.03 g/kg for following 5 days) on soreness, muscle function, and CK activity in resistance-trained males who performed damaging Smith machine squat exercise (5 × 15–20, 50%1RM). Similarly, Machado and colleagues [93] reported that creatine supplementation (1.2 g/kg for 5 days) had no effect on CK levels induced by resistance exercise (five multi-joint exercises, 3 × 10, 75%1RM) in sedentary individuals. McKinnon et al. [94] likewise found no effect of chronic creatine supplementation on soreness or muscle function after eccentric elbow flexion exercise. It is important to note that all of the exercise interventions employed by these studies induced sufficient damage in the participants, as significant perturbations in functional and biochemical outcomes associated with EIMD were reported by the researchers.

Rosene et al. [95] also found no effect of prophylactic (20 g/day for 7 days) creatine supplementation on muscle function and markers of muscle damage in recreationally active males who performed unilateral eccentric knee flexion exercise, though the researchers noted that long-term supplementation (6 g/day for 23 days) preserved post-exercise muscle function compared to placebo following an identical exercise bout in the contralateral leg. Similarly, Cooke and colleagues [96] found that consumption of 0.3 g/kg creatine for 5 days prior to and 0.1 g/kg bodyweight during the 14 days after exercise improved isokinetic and isometric strength and attenuated the CK flux in untrained males who performed intense lower body eccentric resistance exercise. Veggi et al. [97] likewise reported that untrained males who consumed 20 g creatine/day for 6 days between bouts of elbow flexion exercise experienced less soreness and serum CK activity during the 4-day recovery period after the second bout relative to placebo. Creatine has been shown to more consistently attenuate damage induced by endurance or sprint exercise. Short-term dosing of creatine (20 g/day for 5 days) has been shown to reduce inflammation after extended running [98], a half-ironman triathlon [99], and attenuate markers of muscle damage caused by a full-ironman triathlon [100]. Similarly, Deminice et al. [89] reported that post-exercise levels of CRP, TNF-α, and LDH were lower relative to placebo in trained soccer players who consumed 0.3 g/kg creatine for 7 days prior to 12 maximal effort sprints. While mixed results have been reported to date, creatine supplementation shows promise as an effective strategy to mitigate EIMD, provided a sufficient loading dose is consumed (Table 4).

#### β-Hydroxy-β-Methylbutyrate

Early evidence suggests that both acute and chronic consumption of the leucine metabolites β-hydroxy-β-methylbutyrate (HMB) and α-ketoisocaproic acid (KIC) may improve functional and biochemical measures of EIMD, but more information is needed. HMB has been suggested to improve recovery after damaging exercise by increasing protein synthesis and decreasing muscle protein breakdown [101]. Knitter et al. [102] found that 3 g/day HMB supplementation for 6 weeks prior to a 20-km run resulted in lower post-exercise CK and LDH activity in trained male and female runners. van Someren et al. [103] noted that consumption of 3 g HMB and 0.3 g KIC in non-resistance-trained males for 14 days prior to damaging elbow flexion exercise resulted in greater muscle function, lower CK flux, and less soreness at 24 h post-exercise relative to placebo, though a later investigation [104] that employed a similar dosing pattern (3 g HMB and 0.3 g KIC/day for 11 days before and 3 days after exercise) in untrained subjects did not find between-groups differences in muscle function or CK following a 40-min bout of damaging downhill running (a main effect for time was detected for muscle function and CK activity). Similarly, Paddon-Jones and colleagues [105] found no effect of HMB supplementation on EIMD-related outcomes following consumption of 40 mg/kg for 6 days prior to damaging (a main effect for time was detected for post-exercise soreness) eccentric elbow flexion exercise (6 × 4, 30°/s) in non-resistance-trained male subjects. Wilson and colleagues [101] acutely examined the free acid form of HMB (HMB-FA) and reported that consumption of 3 g/day HMB-FA (1 g 30 min before exercise, 1 g during lunch, 1 g during dinner) attenuated the CK response to an acute bout of full-body resistance training. They later reported [106] that 3 g HMB-FA administered prior to damaging isokinetic exercise attenuated LDH but not soreness, muscle function, or CK flux in untrained males, while post-exercise consumption had no effect on relevant outcome measures (Table 4).

#### l-glutamine

Several investigations have shown that consuming at least 0.3 g/kg/day of l-glutamine for up to 3–7 days may improve muscle function and reduce biomarkers of EIMD after damaging exercise. Consumption of supplemental l-glutamine has been suggested to improve immune function and restore plasma concentrations of glutamine, which can decrease markedly during prolonged exercise [107]. For example, Street and colleagues [107] first investigated the effect of glutamine supplementation (0.3 g/kg/day for 3 days) following a bout of drop jumps (5 × 20) in physically active males. The treatment group retained significantly more peak torque and reported less post-exercise soreness compared to placebo over the entire 96-h recovery period. Legault et al. [108] used a similar dosing protocol (0.3 g/kg 1-h prior to exercise, 0.3 g/kg immediately after exercise, 0.3 g/kg/day for 2 days following exercise) in healthy males and females who performed eccentric knee extension exercise (8 × 10, 125%MVC). Glutamine supplementation resulted in reduced post-exercise soreness in both male and female subjects during the entire 72-h recovery period, though only male subjects experienced positive benefits to post-exercise muscle function. A 2016 study [109] likewise found that consumption of 1.5 g/kg glutamine/day for 7 days blunted the CK response to a 14-km run in healthy males. It appears that lower doses of l-glutamine are less effective, as Rahmani Nia et al. [110] found no effect of glutamine supplementation (0.1 g/kg/day for 28 days) on soreness or EMG activity after damaging (main effect for time detected for post-exercise soreness) eccentric leg extensions (six sets to fatigue, 75% 1RM) in untrained males (Table 4).

#### Protein

The effect of protein ingestion on EIMD-related outcomes has been thoroughly reviewed by Pasiakos and colleagues [111] in a 2014 systematic review. In short, the authors concluded that protein supplementation had little effect on muscle damage and noted a large variation in both study designs and outcome measures in the pertinent literature (Table 4).

#### Taurine

Preliminary evidence suggests that the consumption of taurine, an amino-containing sulfonic acid derived from cysteine metabolism, may reduce soreness and improve muscle function after damaging exercise. Taurine exhibits antioxidant effects in vivo and has been suggested to regulate calcium homeostasis in skeletal and cardiac muscle tissues, making it of interest as a potential nutritional adjunct to treat EIMD [112]. Da Silva and colleagues [113] reported that taurine supplementation for 21 days (50 mg/kg/day) resulted in reduced CK and soreness as well as improved muscle function during the 48 h after elbow flexion exercise (3 × 11–15, 80% 1RM) in male subjects. Conversely, Ra et al. [114] found no effect of prolonged taurine use (6 g/day for 14 days prior to and 3 days after exercise) on soreness or markers of muscle damage caused by eccentric elbow flexion exercise (6 × 5, 90% MVC) in untrained men, though a combination of taurine and BCAAs (6 g/day taurine and 9.6 g/day BCAAs) administered via the same supplementation protocol was found to reduce soreness and indirect markers of muscle damage relative to placebo. However, a later study by the same group [115] using an identical taurine supplementation protocol found that soreness during a 4-day recovery period after eccentric elbow flexion (2 × 20, MVC) was attenuated in the recreationally active male participants who consumed taurine. These results were supported by McLeay and colleagues [116], who reported that recreationally active males who consumed taurine (0.1 g/kg/day) for 3 days after a bout of eccentric elbow flexion exercise (6 × 10, MVC) experienced faster recovery of eccentric torque production relative to those who consumed placebo. Thus, taurine supplementation shows promise as a nutritional adjunct to reduce symptoms of EIMD, though more information is needed to determine optimal dosing patterns (Table 4).

### Vitamin Supplements

#### Vitamin C and Vitamin E

The effects of vitamin C and vitamin E supplementation on EIMD have been extensively reviewed in a 2009 article [117] and re-examined more recently by Sousa et al. [12]. Both authors concluded that limited evidence exists to justify the use of supplemental vitamin C and vitamin E to prevent or attenuate muscle damage, as outcomes are consistently mixed in the literature (Table 5).

**Table 5:** Overview of vitamin supplements

Nutrient source (key nutrient)	Mechanism of action	Suggested benefits	Refs
Vitamin C and vitamin E	Antioxidant properties	↓DMG, ↓DOMS, ↓INF, ↑MF	[12, 117]
Vitamin D	Antioxidant properties	↑MF	[118, 123–127]

*↓DMG* nutrient reduced indirect markers of muscle damage compared to placebo, *↓DOMS* nutrient reduced soreness and delayed-onset muscle soreness compared to placebo, *INF* nutrient reduced markers of inflammation compared to placebo, *↑MF* nutrient improved muscle function relative to placebo

#### Vitamin D

Early evidence suggests a potential role of vitamin D supplementation in preventing EIMD, though more evidence is required as mixed results have been reported in the literature. Vitamin D is a secosteroid hormone responsible for skeletal muscle remodeling that has been shown to influence inflammation and muscular function after damaging exercise [118–120]. Vitamin D_3_ (cholecalciferol) is synthesized endogenously upon exposure to ultraviolet-B radiation or consumed in the diet, while vitamin D_2_ (ergocalciferol) is solely found in the diet [121, 122]. Both forms of vitamin D are hydroxylated to the active form of vitamin D (1,24 hydroxyvitamin D) in the liver [122]. Several investigations have examined the interplay between serum vitamin D status and the magnitude of EIMD after eccentric elbow flexion exercise (3 × 12, 75%MVC) [123] and single-leg jumping exercise [124], with conflicting results. Unsurprisingly, relevant interventional studies display similar variability in key outcomes. Barker et al. [125] first demonstrated that vitamin D_3_ supplementation (4000 international units/day [IU/day] for 35 days) prior to eccentric unilateral jumping exercise (10 × 10, 75% body mass) resulted in faster recovery of isometric force relative to placebo in recreationally active males. A later study [118] using a similar prophylactic dosing protocol (4000 IU vitamin D_3_/day for 30 days) in vitamin D-deficient males found similar improvements in muscle function during the recovery period after unilateral eccentric isokinetic exercise (20 × 10, MVC, 30°/s) compared to placebo. Conversely, the first investigation that utilized supplemental vitamin D_2_ derived from portobello mushroom powder (600 IU/day for 6 weeks) found no effect on muscle function or biomarkers of damage after exercise in deficient males [126], while Nieman et al. [127] reported that consumption of 3800 IU vitamin D_2_/day for 6 weeks actually increased the CK and MYO response to a 90-min eccentric exercise bout (consisting of 17 different multi-joint exercises) relative to placebo in trained NASCAR pit crew staff. The authors noted that serum 25-hydroxyvitamin D_2_ concentration, a marker of vitamin D_2_ status, was significantly elevated, while serum 25-hydroxyvitamin D_3_, a marker of vitamin D_3_ status, was significantly decreased after 6 weeks of vitamin D_2_ supplementation and suggested that the negative effects of the supplement may have been caused by the combined effect of elevated vitamin D_2_ and reduced vitamin D_3_ status. Thus, it appears that supplemental vitamin D_3_ may improve EIMD-related outcomes, while supplemental vitamin D_2_ may increase the magnitude of EIMD relative to placebo (Table 5).

### Other Supplements/Nutritional Strategies

#### n-3 Polyunsaturated Fatty Acids

Much of the pertinent literature suggests that the prophylactic consumption of n-3 polyunsaturated fatty acids (n-3 PUFAs) may improve a variety of EIMD-related outcomes, though variability in study designs, dosing protocols, and outcome measures make comparisons challenging. n-3 PUFAs such as eicosapentaenoic acid (EPA) and docosahexaenoic acid (DHA) have been shown to modulate inflammation and immune function, and have been suggested to play a role in the regulation of skeletal muscle protein synthesis [119]. Several studies have reported beneficial effects of prophylactic n-3 PUFA consumption on post-exercise soreness in males [128–130], females [131, 132], and in mixed-gender cohorts [133, 134] at dosages that generally have ranged from 0.54 to 3 g/day for 7–60 days. Tartibian et al. [128] first reported that consumption of 324 mg EPA and 216 mg DHA per day for 32 days reduced perceived pain and range of motion decrements 48 h after exercise (40 min of bench stepping exercise using a 50-cm step) performed after 30 days of supplementation in healthy males. Similarly, Tsuchiya and colleagues [130] noted that healthy males who consumed 600 mg EPA and 260 mg DHA per day for 8 weeks prior to and 5 days after unilateral isokinetic elbow flexor exercise (5 × 6, MVC, 90°/s) experienced less soreness after 2 days of recovery and displayed improved muscle function relative to placebo during days 2–5 of the recovery period. Philpott and colleagues [129] likewise demonstrated that 1100 mg EPA and 1100 mg DHA added to a whey protein supplement that was consumed by competitive male soccer players each day for 6 weeks prior to 12 sets of eccentric knee extension exercise resulted in significantly decreased soreness during 72 h of recovery compared to consumption of the whey protein supplement alone. Finally, Mickleborough [135] found that untrained males who consumed a proprietary supplement containing 58 mg EPA and 44 mg DHA for 26 days before and 4 days after a bout of downhill running exhibited significantly less TNF-α and CK flux at all time points during the 4-day period after exercise. Furthermore, the treatment group also reported significantly less soreness and retained greater muscle function at 96 h of recovery.

Several investigations have also reported beneficial effects of n-3 PUFA supplementation in female participants. Corder and colleagues [131] noted that 9 days of DHA consumption (3000 mg/day) reduced the increase in soreness caused by eccentric elbow flexion exercise (four sets to fatigue, 120%1RM) across 48 h of recovery in healthy, untrained female participants. Similarly, Tinsley et al. [132] found that healthy, non-resistance-trained females who consumed 3000 mg EPA and 600 mg DHA per day for 1 week prior to and 1 week after elbow flexion and knee extension exercise (ten sets to fatigue, 50%1RM) reported less muscle soreness at 48 h post-exercise, though these differences were not statistically significant. Jouris and colleauges [133] also reported that 7 days of 2000 mg EPA and 1000 mg DHA supplementation in healthy men and women blunted post-exercise soreness at 48 h of recovery that was induced by two sets of elbow flexion exercise to fatigue at 120%1RM. Lembke et al. [134] likewise found that 30 days of n-3 PUFA supplementation (2.7 g/day) prior to eccentric forearm extension exercise (2 × 30) resulted in significantly decreased soreness 72 h after exercise compared to placebo in healthy male and female participants.

However, several studies [136–138] have found little to no effect of n-PUFA ingestion on functional or biochemical outcomes associated with EIMD. Lenn and colleagues [136] reported that consumption of 1.8 g n-3 PUFAs per day for 37 days was not sufficient to impact EIMD in healthy males and females who performed 50 maximal isokinetic elbow flexion contractions (sufficient damage was induced, as a main effect for time was observed for muscle function). Bloomer et al. [138] also found no effect of 6 weeks of n-3 PUFA consumption (2224 mg EP and 2208 mg DHA per day) on markers of muscle damage induced by a 60-min treadmill climb. However, the protocol did not induce sufficient damage in the well-trained participants. Gray and colleagues [137] likewise reported that consumption of 1300 mg EPA and 300 mg DHA per day had no effect on EIMD-related outcomes in recreationally trained males, even though sufficient muscular damage was induced (a main effect for time was observed for soreness at 48 h post-exercise). Finally, Houghton and Onambele [139] reported a 20% increase in IL-6 flux compared to placebo after damaging resistance exercise in spite of prophylactic n-3 PUFA supplementation (360 mg/day for 21 days). The authors theorized that these results may have been caused by increased EPA-mediated contractile capacity, resulting in greater glycogen depletion, glucose metabolism, and elevated IL-6 levels. Regardless, the divergent results of the many studies outlined in this section may be explained in part by differences in exercise modality and intensity as well as supplement dose and duration. Consequently, more work is needed in this area (Table 6).

**Table 6:** Overview of other nutritional supplements

Nutrient source (key nutrient)	Mechanism of action	Suggested benefits	Refs
n-3 PUFAs (*DHA*, *EPA*)	Anti-inflammatory properties	↓DMG, ↓DOMS, ↓INF, ↑MF	[128–139]
Caffeine	Adenosine receptor antagonism	↓DOMS	[141–147]
MSM	Antioxidant properties	↓DMG, ↓INF	[148–150]
MIPS (*Caffeine*, *Creatine*, *BCAAs*)	Increased MPS	NA	[17, 152]
Specialized water supplements	Anti-inflammatory properties	↓DMG, ↓DOMS, ↓INF, ↑MF	[153–157]

*DHA* docosahexaenoic acid, *↓DMG* nutrient reduced indirect markers of muscle damage compared to placebo, *↓DOMS* nutrient reduced soreness and delayed-onset muscle soreness compared to placebo, *EPA* eicosapentaenoic acid, *↓INF* nutrient reduced markers of inflammation compared to placebo, *MIPS* multi-ingredient pre-workout supplements, *↑MF* nutrient improved muscle function relative to placebo, *MSM* methylsulfonylmethane, *NA* nutrient had no effect on EIMD-related outcome measures, *n-3 PUFAs* omega-3 polyunsaturated fatty acids

#### Caffeine

The acute pre-exercise consumption of moderate doses of caffeine appears to ameliorate soreness caused by damaging exercise, though this strategy has little effect on indirect markers of muscle damage. Caffeine is an adenosine receptor antagonist and possesses antinociceptive effects, thus acutely reducing the perception of pain [140]. In a series of studies, Machado and colleagues [141–144] showed the acute consumption of a moderate dose of caffeine (4.5–5.5 mg/kg) approximately 45 min before exercise had no effect on serum CK and LDH after sprint exercise (6 × 10, 20 m sprints) in male soccer players. Similarly, Vimercatti et al. [145] found no effect of acute caffeine consumption (4.5 or 5.5 mg/kg) on the CK and LDH flux following treadmill exercise (65% VO_2_max) in physically active males. However, Hurley and colleagues [146] reported that resistance-trained males who ingested 5 mg/kg caffeine 1 h before elbow flexion exercise (5 × 10, last set to fatigue, 75%1RM) reported significantly lower soreness during days 2 and 3 of a 5-day recovery period compared to placebo. Similarly, Caldwell et al. [147] showed that trained cyclists who consumed 3 mg/kg caffeine immediately after a 164-km cycle race and for the next four mornings reported significantly lower leg muscle soreness during the afternoon data collection sessions conducted during three consecutive days of post-exercise recovery compared to placebo. Thus, the analgesic effects of caffeine appear to be the primary benefit of this strategy (Table 6).

#### Methylsulfonylmethane

Several investigations have reported beneficial effects of the sulfur-containing molecule methylsulfonylmethane (MSM) on EIMD. MSM has been demonstrated to possess anti-inflammatory and antioxidant effects [148]. Barmaki et al. [149] noted that the chronic consumption of MSM (50 mg/kg/day for 10 days) attenuated the CK response to a 14-km run in healthy young men. Similarly, van der Merwe and colleagues [150] found a dampened post-exercise cytokine response in physically active males who consumed 3 g MSM/day for 28 days prior to and 3 days after eccentric knee extension exercise (1 × 10, 100%1RM). However, Withee et al. [148] found no effect of prophylactic MSM supplementation (3 g/day for 21 days) on muscle pain, joint pain, or markers of damage in trained runners after a half-marathon race. Clearly, more work is required before MSM can be conclusively recommended as an efficacious strategy to reduce EIMD (Table 6).

#### Multi-Ingredient Pre-Workout Supplements

Preliminary evidence shows little effect of prophylactic consumption of multi-ingredient pre-workout supplements (MIPS) on EIMD-related outcomes. Due to the varied ingredient profile of these supplements and prevalence of proprietary blends, the efficacy and mechanism of action can vary substantially between individual supplement formulations [151]. Ormsbee et al. [152] found no effect of 31 days of MIPS consumption (21 g/day) on functional and biochemical measures of EIMD after downhill running in trained male runners. A later study by the same group [17] using a cohort of trained female runners found that prophylactic consumption of the same MIPS (21 g/day for 28 days) likewise had no effect on markers of damage after a similar exercise stimulus. However, further research should be undertaken using different formulations and exercise stimuli before results are generalized further (Table 6).

#### Specialized Water Supplements

Early evidence suggests that consumption of several types of specialized water supplements may reduce soreness and improve muscle function by improving post-exercise hydration [153], though more information is needed regarding this approach. Hou and colleagues [154] investigated the effect of post-exercise deep ocean mineral water consumption (150% lost body mass) on markers of muscle damage following a bout of fatiguing treadmill exercise (40%VO_2_max performed until 3% body mass was lost) and found that those in the treatment group retained more muscle function and exhibited lower CK and MYO activity during the recovery period. These results were supported by two later studies which found that the post-exercise consumption of deep ocean [153] and bedrock-sourced [155] mineral water improved muscle function following prolonged, dehydrating aerobic exercise. Similarly, Borsa and colleagues [156, 157] found that the consumption of a bodyweight-dependent dose of electrokinetically modified water for 18 days before and 4 days after elbow flexion exercise (3 × 20) attenuated post-exercise CK and CRP activity, preserved muscle function, and reduced pain during a 96-h recovery period relative to placebo in resistance-trained males and females (Table 6).

### Practical Considerations And Future Directions

The decision to implement nutritional strategies to attenuate the effects of EIMD must be made with the potential negative effects of these strategies in mind, as the chronic use of many of these nutrients may impair training adaptations [11]. However, maximizing recovery capacity at the expense of long-term training adaptations may be desirable in athletes who need to recover as quickly as possible from the demands of training or competition. Similarly, the application of chronic/prophylactic nutritional or supplementation strategies in conjunction with a periodized training program may facilitate peaking/overreaching strategies and allow for increased training volume during these phases. Finally, reducing the symptoms of EIMD may improve exercise compliance in untrained individuals [132] or older adults [158] who might perceive muscular stiffness or soreness as a negative outcome when beginning an exercise program. Decisions regarding which nutritional strategy to employ should also be made with the other physiological effects of the substance in mind. For example, endurance athletes who require enhanced recovery from damaging exercise would likely benefit in multiple ways from choosing beetroot juice as a recovery strategy, as beetroot juice acts on EIMD-related symptoms [50, 52] and also improves endurance exercise performance when consumed chronically [159]. Similarly, a strength athlete seeking an effective nutritional recovery modality would benefit from the well-supported ergogenic effects of chronic creatine consumption [90] in addition to the substance’s effect on EIMD [96, 97].

Future research in this area should investigate the combined effects of multiple nutritional or supplementation strategies on EIMD-related outcomes. It is possible that prophylactic supplementation with sufficient doses of multiple nutrients that act on different physiological mechanisms could result in a synergistic effect on symptoms of EIMD beyond that of single nutrients alone. For example, a combination of sufficient doses of tart cherry juice (which confers antioxidant effects [160]), curcumin (which influences COX-2 signaling [68]), β-hydroxy-β-methylbutyrate (which improves muscle protein synthesis [101]), and vitamin D_3_ (which regulates skeletal muscle function and inflammation [120]) may facilitate recovery from EIMD in a manner that is greater than any single supplementation strategy on its own. In addition, more information is required regarding the interplay between the nutritional/supplementation strategies and other treatment options for EIMD, such as massage or cryotherapy.

## Conclusions

Because exercise-induced muscle damage can cause significant discomfort and impair subsequent athletic performance or training quality, the development of effective nutritional and supplementation strategies to combat EIMD is of paramount importance to athletes, coaches, dieticians, researchers, and fitness professionals alike. This review outlined a wide assortment of ingredients, functional foods, and dietary supplements that have been shown to affect EIMD-related outcomes, with varying levels of success. In addition to the topics covered in the present review, many emergent nutritional and supplementation strategies have not been fully explored, including black tea-sourced polyphenols [161], blueberries [162], chondroitin sulfate [163], high chlorogenic acid coffee [164], fasting [165], garlic [166], leucine metabolites such as HICA [167], lemon verbena [168], lychee [169], mate tea [170], pequi fruit [171, 172], quercetin [173], saffron [174], selenium [175], sesame [176], spinach [177], and tomato juice [178, 179]. Future research in these nascent areas may shed new light on potential treatment options for EIMD.

## Abbreviations

1RM: One-repetition maximum
BCAA: Branched chain amino acid
CK: Creatine kinase
CRP: C-reactive protein
EIMD: Exercise-induced muscle damage
HMB: β-Hydroxy-β-methylbutyrate
HMB-FA: β-Hydroxy-β-methylbutyrate free acid
IL-6: Interleukin 6
IU: International unit
KIC: α-Ketoisocaproic acid
LDH: Lactate dehydrogenase
MHR: Maximal heart rate
MIPS: Multi-ingredient pre-workout supplements
MRI: Magnetic resonance imaging
MSM: Methylsulfonylmethane
MVC: Maximal voluntary contraction
MYO: Myoglobin
n-3 PUFAs: n-3 polyunsaturated fatty acids
NSAIDS: Non-steroidal anti-inflammatory drugs
PA: *Phyllanthus amarus*
PPT: Pressure pain threshold
RR: *Rhodiola rosea*
TNF-α: Tumor necrosis factor-alpha
VO_2_max: Maximal oxygen consumption
VO_2_peak: Peak oxygen consumption
